# Principles of Eugenics

**Published:** 1915-02

**Authors:** Malone Duggan


Principles of Eugenics.—A Practical Treatise. By Blanch Eames. Moffat, Yard & Company.

This is a cleverly written little volume purporting to discuss the principles of eugenics. The moral tone is high, and a definite purpose is imminent in every chapter. The object uppermost seems to be to teach the author's idea of sex morality. She quotes largely from the best scientific authorities, and her data is so well correlated that to an indisciminent reader it might pass as accepted facts. The book is simply another instance of stating a proposition based on a theological principle and bending and warping scientific facts to substantiate the contention. But for the harm and added misery to humanity that might come from accepting the doctrine it teaches on continence it might pass as a very readable and helpful book. MALONE DUGGAN.

